# From Transcriptomics to Treatment in Inherited Optic Neuropathies

**DOI:** 10.3390/genes12020147

**Published:** 2021-01-22

**Authors:** Michael James Gilhooley, Nicholas Owen, Mariya Moosajee, Patrick Yu Wai Man

**Affiliations:** 1 Institute of Ophthalmology, University College London, Bath Street, London EC1V 9EL, UK; n.owen@ucl.ac.uk (N.O.); m.moosajee@ucl.ac.uk (M.M.); py237@cam.ac.uk (P.Y.W.M.); 2 Moorfields Eye Hospital NHS Foundation Trust, 162 City Road, London EC1V 2PD, UK; 3 The Francis Crick Institute, 1 Midland Road, Somers Town, London NW1 1AT, UK; 4 Great Ormond Street Hospital for Children NHS Foundation Trust, London WC1N 3JH, UK; 5 Department of Clinical Neurosciences, University of Cambridge, Robinson Way, Cambridge CB2 0PY, UK; 6 MRC Mitochondrial Biology Unit, University of Cambridge, Robinson Way, Cambridge CB2 0PY, UK; 7 Cambridge Eye Unit, Addenbrooke’s Hospital, Hills Road, Cambridge CB2 0QQ, UK

**Keywords:** transcriptomics, RNA-seq, neuroprotection, DOA, OPA1, LHON, gene-therapy, mitochondrial, optic neuropathies

## Abstract

Inherited optic neuropathies, including Leber Hereditary Optic Neuropathy (LHON) and Dominant Optic Atrophy (DOA), are monogenetic diseases with a final common pathway of mitochondrial dysfunction leading to retinal ganglion cell (RGC) death and ultimately loss of vision. They are, therefore, excellent models with which to investigate this ubiquitous disease process—implicated in both common polygenetic ocular diseases (e.g., Glaucoma) and late-onset central nervous system neurodegenerative diseases (e.g., Parkinson disease). In recent years, cellular and animal models of LHON and DOA have matured in parallel with techniques (such as RNA-seq) to determine and analyze the transcriptomes of affected cells. This confluence leaves us at a particularly exciting time with the potential for the identification of novel pathogenic players and therapeutic targets. Here, we present a discussion of the importance of inherited optic neuropathies and how transcriptomic techniques can be exploited in the development of novel mutation-independent, neuroprotective therapies.

## 1. Introduction

### 1.1. Optic Neuropathies

Optic neuropathies are among the most common causes of blindness in the working age population [1], with inherited forms (including Leber Hereditary Optic Neuropathy (LHON) [2] and Dominant Optic Atrophy (DOA)) affecting about 1 in 10,000 of the population [3,4,5]. In this review, we will present a brief introduction to these conditions and how the availability of powerful emerging techniques, including transcriptomics, are quickly revolutionizing both diagnosis and development of novel therapies with potential applications beyond the eye.

Vision is ultimately lost in both LHON and DOA as retinal ganglion cells (RGCs) die secondary to mitochondrial dysfunction [6]. This specific susceptibility of RGCs to such dysfunction is not completely understood. However, the relatively large metabolic demand for these specialized cells and their unique anatomy are thought to be important contributory factors. RGCs have long axonal segments which lack myelin throughout their intraocular course but gain a myelin sheath on exiting the eye beyond the lamina cribosa [7,8]. As the only nervous tissue visible *in vivo* and with increasingly sophisticated cell culture techniques [9,10], RGCs present a powerful system in which to interrogate mitochondrial dysfunction and the pathways that ultimately lead to cell loss and disease development. Such dysfunction has been implicated in major neurodegenerative diseases, such as Parkinson’s disease [11], Alzheimer’s disease [12], and other forms of dementia [13], but the polygenetic inheritance and environmental contribution to these common conditions are particularly challenging when investigating their pathogenesis.

As monogenetic conditions where mitochondrial function is disturbed, both LHON and DOA can mitigate some of these challenges and act as useful model diseases of more complex neurodegenerative disease processes. LHON is a primary mitochondrial DNA (mtDNA) disorder, with the majority of cases caused by one of three point mutations—namely, m.3460G>A in the *MT-ND1* gene, m.11778G>A in the *MT-ND4* gene, and m.14484T>C in the *MT-ND6* gene—all of which encode for essential subunits of mitochondrial complex I [3,7,14,15,16]. LHON is additionally interesting due to its predilection to manifest in males and its marked incomplete penetrance—both of which could perhaps have origins in transcriptomic differences.

In comparison, DOA is nuclear-encoded mitochondrial optic neuropathy caused by mutations in the *OPA1* gene (3q28-q29), which encodes for a multimeric dynamin GTPase protein located within the mitochondrial inner membrane. *OPA1* subserves a number of functions, including the regulation of mitochondrial fusion, the stability of the mitochondrial respiratory chain complexes and mitochondrial biogenesis, the sequestration of pro-apoptotic cytochrome *c* within the mitochondrial cristae, and mitochondrial turnover (mitophagy) [4,17,18,19,20].

### 1.2. “-Omics” Technologies as Applied to Inherited Optic Neuropathies

Our understanding of mitochondrial biology and disease has advanced greatly over recent years, not least due to the development and maturation of “-omics” technologies. These can be defined as “high-throughput technologies capable of detecting differences in a multitude of molecular constituents in organisms [21]”, with those that represent the three strata of central biological dogma (gen*omics*, transcript*omics*, and prote*omics*) being prominent. These fields deal with the detection of differences in DNA sequences, gene transcription, and proteins within tissue. Additionally, particularly relevant to mitochondrial disease is the developing field of metabol*omics* (centered on the comparison of levels of products of metabolism) [22,23] and lipid*omics* [24].

**Figure 1:**
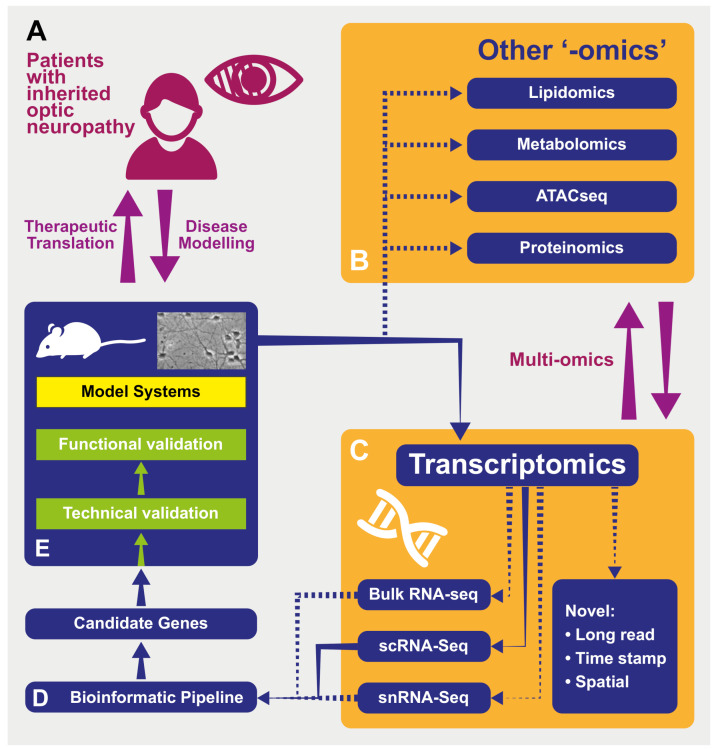
A schematic representing the processes of modeling and investigating inherited optic neuropathies using “-omics” methods. (**A**) Model diseases such as DOA and LHON can be powerful tools in which to investigate the effects of mitochondrial dysfunction. The study of patients with inherited optic neuropathies is often a two-way process: in one direction, the characterization of their phenotype and genotype allows the development of useful disease models (for example, cell -and animal-based). These provide an efficient environment in which to increase our understanding of underlying disease processes as well as a testing ground for novel therapies before their return in the opposite direction, back into patients. (**B**) Tissues from model systems can be investigated using used in multiple “-omics” techniques—in some cases, these can be performed simultaneously and analyzed in vertical “multi-omics” experiments (see text). ATACseq—Assay for Transposase Accessible Chromatin—a method for assessing which areas of the chromatin superstructure are open and so likely available for active in transcription, is an example of the rapidly expanding field of *epigenomics*. (**C**) Multiple transcriptomic techniques are available and have particular strength in different experimental situations. “Novel”: Novel techniques are emerging that will further the resolution of these techniquesm including those with abilities to sequence longer transcripts in one read and those that integrate temporal and spatial information regarding transcripts (see text). RNA-seq—RNA-sequencing; scRNA-seq—single-cell RNAseq; snRNAseq—single nucleus RNAseq. (**D**) Transcriptomic techniques gain power from the large quantity of data that they produce. This necessitates adequately designed bioinformatic pipelines that are tailored to the exact scientific question being asked in order to produce a list of candidate genes for further investigation back in model systems. (**E**) Model systems of diseases can include those based on cultured patient cells or be animals carrying pathogenic variants, leading to phenocopies of human disease. Samples from these models used in transcriptomic analysis can include tissue (such as retinas) or cell cultures. As many transcriptomic experiments compare expression between conditions (e.g., disease and control) to produce lists of differentially expressed genes, the further technical and functional validation of these can be performed back in model systems in preparation for therapeutic translation in patients (see text).

Whilst these disciplines are linked by their comparative nature—experimental plans often involve the contrast of different conditions (e.g., control and “diseased” states, or between different cell types)—the emerging field of multi-omics (or vertical -*omics*) focuses on complimentary comparisons *across* domains (Figure 1). For example, highlighting changes replicated across the transcriptome, the proteome and metabolome will carry particular significance [24,25], and this approach is already being used in mitochondrial research [26]. As these technologies and their complementary bioinformatic analysis techniques develop, the power of “-*omics*” investigations is likely to increase.

### 1.3. Transcriptomics

Specifically, “the transcriptome” refers to the RNA transcribed within a cell, or group of cells, often with a particular focus on mRNA (both coding and non-coding). Several methods to quantify mRNA have been developed (see below) and are applied to genetic eye diseases. For example, assessing transcribed features in a particular sample can be used to compare changes in gene expression either over time or between control and diseased states (such as optic neuropathies) [27]. Features showing differential expression may be implicated in the disease process, highlighting areas for further investigation to uncover aetiologic pathways, novel biomarkers, and therapeutic targets.

## 2. Transcriptomics in Inherited Optic Neuropathies

### 2.1. Applications of Transcriptomics in Optic Neuropathies

Several factors make transcriptomics a particularly suitable methodology for the investigation of optic neuropathies. Whilst the anatomy and physiology of the retina and optic nerve are relatively well understood compared with other areas of the central nervous system [28], our understanding of pathophysiology in these structures can be less comprehensive. This is particularly true for inherited optic neuropathies such as LHON and DOA, where the genomic determinant of the disease in many patients is readily identifiable as a single gene (monogenic disorder) [14,18,19]. However, less is known regarding how this translates into the clinical phenotype of RGC death as well as other as yet unexplained facets of these model diseases (such as the incomplete penetrance in LHON when the pathogenic mitochondrial DNA mutation is present in the homoplastic state in both affected patients and carriers). Thus, this presents an unmet need to identify the novel pathways and genes involved to which comparative transcriptomics is particularly suited. Whilst direct access to RGCs and patient tissues is limited, cellular and mouse model systems [7,29,30] have developed in recent years into powerful platforms with which to perform transcriptomic studies (and, more importantly, validate and investigate their findings). For example, *in vivo* neuro-retinal tissue can easily be visualized (if not directly sampled) at the cellular level with techniques such as optical coherence tomography (OCT), and there are well defined metrics of RGC function at the behavioral (acuity) [31], reflex (pupillary) [32], and electrophysiological levels [33]. To compliment this, induced pluripotent stem cell (iPSC) RGC models derived from LHON and DOA patient tissues have proved invaluable for molecular investigations [7]

### 2.2. Disadvantages of Transcriptomics in Optic Neuropathies

Despite the suitability of inherited optic neuropathy investigations, the limitations of transcriptomics must be borne in mind when considering data from such studies. In isolation, transcriptomics gives us no direct information on protein dynamics. The mRNA expression level of a particular gene may correspond to increased protein levels, increased protein turnover, or indeed changes to post translational protein modifications. Therefore, the validation of transcriptomic findings at the protein or functional level is required if conclusions are to be drawn regarding the downstream effects of mRNA changes. Planning this can present further challenges—for example, when comparing transcriptomes in conditions (such as optic neuropathies) with changes dramatic enough to lead to cell death, large numbers of differentially expressed genes (DEGs) are likely. Therefore, methods to prioritize which DEG to validate while minimizing bias have been developed, and these are discussed further below and reviewed elsewhere [34].

Whilst preparing tissue for transcriptomic studies, careful tissue handling is equally important in reducing bias, and indices of extracted RNA quality (such as RNA Integrity Number “RIN” [35]) can be used to assess this. Additionally, and especially in a highly cellular, complex tissue such as the retina, it is essential to ensure that the identity of cells undergoing processing is known (for example, photoreceptors and bipolar cells have an interdigitated synapse that can make them difficult to dissociate and isolate [36]. Additionally, the cells isolated must be viable. It is well established that the dissection of retina from mouse models requires the cutting of the optic nerve (and therefore the transection of RGC axons), so processing should be as expedient as possible to minimize the stress response recorded. Indeed, many of these limitations have been addressed as isolation methods have been developed.

## 3. Transcriptomic Methodologies

### 3.1. Tissue Selection and Sample Preparation

One methodological aspect that has been particularly refined is that of tissue selection and preparation. Earlier studies made use of tissues dissected by hand from animal models [37] and dissociated into suspension or collected from cultured cells [38] in bulk. This has the advantage of providing a large quantity of RNA for further processing. In addition, the processing of tissue in this way is relatively easy and expedient, reducing postmortem alterations in expression—particularly relevant to RGCs following the truncation of their axon during enucleation. Indeed, while improvements have been made to tissue preparation processes (see below), the basic processes remain constant: RNA is extracted, isolated, and reverse-transcribed into complementary DNA (cDNA) representing a *library* of the transcripts present [27]. Libraries are sequenced after undergoing amplification [39] or enrichment steps for a particular form of RNA—for example, selecting for the poly-adenosine tail of transcribed mRNA (of either nuclear or mitochondrial origin) [40] or depleting ribosomal RNA before reverse transcription [41] in order to focus on protein coding mRNA.

### 3.2. Quantifying Expression

#### 3.2.1. Quantitative Reverse Transcription Polymerase Chain Reaction (qRT-PCR)

Once the library has been prepared, it is necessary to quantify the expression of transcripts. In the most basic form, qRT-PCR is an accurate technique to interrogate a small number of genes [42,43]; however, it is limited by its need for oligo-primers requiring the *a priori* selection of candidate genes. This technique remains in use as a confirmatory tool (for example, to confirm sample purity) due to its ease, ubiquity, and economy. However, the inherent amplification of error in the technique [44,45] and the concurrent improvement in the technical reliability of microarray and RNA-seq techniques has seen it widely replaced by other assays in validation experiments.

#### 3.2.2. RNA-Seq

Whilst the development of microarray assays [46] represented a paradigm shift and the beginning of high-throughput transcriptomics, their requirement to define which genes to investigate *a priori*, as well as technical limitations [47,48], have led to their replacement in most applications by RNA-seq techniques. RNA-seq refers to techniques where cDNA is directly sequenced using high-throughput sequencing techniques [27]. The superior flexibility and unbiased nature of this approach (not requiring the *a priori* definition of probes) has seen it supersede microarrays in many areas—particularly comparative transcriptomics. Indeed, due to these advantages RNAseq has become an integral technique in clinical and laboratory science and is the the gold standard in multiple disciplines, most notably in oncology [49] but also more generally (reviewed elegantly here [50]).

Several modifications of this technique are particularly relevant to the study of RGCs and optic neuropathies. Given the diversity of RGC cell types [51,52,53] and their differential response to disease [54], techniques that are able to prepare libraries from and sequence the transcriptomes of individual cells (single cell "scRNA-seq” [55,56] or single-nuclei snRNA-seq [57]), are particularly useful. These techniques overcome difficulties with “*bulk*” RNAseq techniques in resolving changes in gene expression between subpopulations of cells. This is achieved by labelling individual transcripts as being from a particularly labelled cell: this vastly increases resolution by allowing analysis at the level of individual cells, but concurrently increases the complexity of analysis and the resources required.

In such methods, single cells are isolated (for example, using a microwell plate or in individual droplets using microfluidics) and combined with reaction substrate and a barcoded bead (see [58] for a review of individual methods). This allows library preparation and sequencing on a cell by cell basis, potentially uncovering changes masked by techniques dealing with bulk batches of cells [59]. Single-cell sequencing has also been useful when used with *clustering* techniques to group similar single-cell transcriptomes from individual RGCs in the search for novel molecular markers correlating with anatomical and functional characteristics for subpopulations of RGCs [53].

#### 3.2.3. Future Directions in Quantifying Expression

This characterization of RGC subpopulations and their role in optic neuropathies is likely to particularly benefit from rapidly developing new technologies within RNA-seq. Improvements in the resolution of the single-cell “long read” sequencing [60] (as opposed to short reads focusing on the 3′ end of transcripts) and RNA timestamping [61] (which details temporal changes in mRNA transcripts) have enhanced our ability to understand how differing transcripts interact and turn over. At the tissue level, the emergence of technology to allow single-cell multiomics simultaneously on the same cells [62] will further increase the resolution of our understanding of these cells in optic neuropathies.

Perhaps most excitingly, however, is the emerging technique of spatial transcriptomics [63], in which the molecular labelling of individually sequenced cells can preserve information regarding their anatomical location. If this can be developed in conjunction with multiple electrode array neurophysiology [64], which similarly preserves retinal topology, there is potential for a technique to obtain both single-cell transcriptomic and single-cell functional data from the same cells, allowing direct validation.

### 3.3. Analysis of RNA-Seq Data

As RNA-seq techniques have developed over the past few years, so too have the bioinformatic approaches required to make sense of the often vast quantities of data produced. Indeed, there is no “gold-standard” pipeline or set of processes for either bulk [65] or scRNA-seq [66] analysis, and the approaches have to be tailored to the individual experiment and the scientific question being addressed by a particular study. However, general stages of processing do tend to be applied, even if their details vary (Figure 1, and reviewed here [27,55,67,68,69]). This variability and the great pace of advancement in bioinformatic methodologies make it essential to precisely document software versions and the settings, as these can greatly alter results [55]. Indeed, replicating notable results using different software packages may add to the power of conclusions.

## 4. Clinical and Research Applications of Transcriptomics

### 4.1. Diagnostic

Mirroring this rapid advancement in data analysis and software techniques in RNA-seq has been a concurrent improvement in our diagnostic capabilities in genetic disease. In recent years, approaches to attaining molecular diagnoses in optic neuropathies and in genetic disease more generally have changed with the continually improving technologies. The traditional approach of testing for a particular pathogenic variant in a single gene based on a characteristic clinical presentation has been extended with the testing of “panels” of genes associated with a particular phenotype (optic atrophy, for example). This has the advantage of combining clinical *prior probability* to provide a high diagnostic confidence when positive, but limits diagnoses to those pre-determined variants tested for on the panels. While whole-exome (WES) and whole-genome sequencing (WGS) have increased diagnostic yields up to as much as 50% of cases [70] by allowing the identification of novel variants, one of their disadvantages is not providing direct information on the pathogenicity of variants [71].

In this situation, sequencing transcribed RNA (RNA-seq) can be hugely useful in indicating the downstream effects of variants—for example, by detecting expression levels outside the normal physiological range (up or down), splicing-related errors, posttranscriptional modifications, or mono-allelic expression [72]. This is especially useful where little is known regarding a gene, its role in the target tissue, or variants in non-coding regions. In combination with WGS, RNA-seq has been used to characterize novel disease-causing genes in mitochondrial disease [73].

One potential drawback to diagnostic RNA-seq in optic neuropathies lies in its need to be performed on biopsied disease tissues where the target genes are expressed, which is not feasible for the optic nerve. A muscle biopsy has proven to be a useful surrogate in mitochondrial disease, given its high energy demand [74]. However, it is emerging that many genes can be investigated using less invasive techniques, such as blood sampling [74]. With the licensing of gene replacement therapies, the need for molecular diagnosis is more important than ever for individual patients in order to select the most appropriate gene therapy as these options emerge. To this end, RNA-seq is an important addition to our armamentarium by expanding our diagnostic ability.

### 4.2. Disease Mechanisms

#### 4.2.1. Exploring Disease Mechanisms Using Transcriptomics

As discussed above, transcriptomics is invaluable to the identification of differentially expressed genes between samples, in particular the comparison of healthy and diseased tissues. This has great potential for inherited optic neuropathies (see Table 1) where the mechanisms connecting pathogenic genomic variants and eventual RGC death are poorly understood. Genes expressed at significantly higher or lower levels in diseased tissues compared to healthy tissues are obvious candidate players involved in the underlying disease process [37]. However, with the scale of transcriptomic data, this list can extend to many hundreds of genes, and processes are required to prioritize the most biologically plausible candidates for further investigation.

A fruitful approach to this has been to interrogate lists of differentially expressed genes (DEGs) for *connected* sets of genes. With the development of microarray and RNA-seq technology [75], increasingly sophisticated methods, both those relying on the intrinsic properties of DEG data sets (“unbiased”) and those drawing from databases of gene and protein function and interactions [76,77,78,79] (introducing “biological insight”) have been developed. These include overrepresentation analysis, where lists of DEGs are compared to annotated databases (such as Gene Ontology [80] categories) to highlight ontologies that are seen particularly often in the list [76]. Gene Set Enrichment Analysis (GSEA) [81] provides another strategy where lists of DEG are interrogated for the overrepresentation of groups of genes that are connected to particular biological functions. Ultimately, however, the decision on which DEGs to further investigate in order to best answer their individual research question lies with the investigator, armed with the objective prioritization that these techniques allow (Figure 1).

#### 4.2.2. Technical Validation

Having prioritized candidate DEGs for further investigation, initial investigations are needed to validate the transcriptomic findings at the technical level [90]. This can involve techniques to confirm sample purity, such as the qRT-PCR of genes that are known to be expressed (or not) in the sampled tissue. For example, in a sample of RGCs this involves checking that known RGC-specific genes (such as *THY1*) are expressed and those known to be specific to other, potentially contaminating, cell types (such as *RHO* from rods) are absent [36,44,45]. In addition, confirming that proteins relating to candidate genes are present in target tissues (for example, by immunohistochemistry, Western blotting, or similar) is a helpful approach to relate findings at the transcriptomic to the effector (protein) level if the appropriate *a priori* knowledge is available.

#### 4.2.3. Functional Validation

When investigating potential disease mechanisms in optic neuropathies, the validation of the functional role of DEGs is paramount. Do changes identified at the transcriptomic level result in an alteration in cellular function relative to disease progression which could represent a therapeutic target? As direct access to retinal and optic nerve tissues from patients with inherited optic neuropathies is not practical, such work has relied heavily on preclinical models. These have been established from patient-derived cells (blood cells, fibroblasts, lymphoblasts, and cybrid cell lines) which allow the faithful replication of some cellular disease processes found in mutation-carrying cells, but outside of the RGCs themselves (extensively reviewed elsewhere [10]). Over the last decade, technological advances have allowed the generation of RGC cell lines [9] engineered from patient fibroblasts (via induced pluripotent stem cell technology) and animal disease models [7] that faithfully replicate the environment of the RGC in the diseased human retina (reviewed in [7]).

Within model systems, the overexpression or inhibition of candidate genes can be fruitful approaches to validation. In the context of a promising therapeutic target, the manipulation of levels of gene expression in the model should lead to in changes in the metrics of the disease process (as in [59]). For an inherited optic neuropathy, the parameters can be anatomical, such as the rate of RGC survival, or relate to a known pathological process contributing to disease (e.g., mitochondrial oxidative phosphorylation or reactive oxygen species turnover [91]).

The increasing variety and sophistication of *in vitro* and *in vivo* disease models for inherited optic neuropathies offer differing advantages that could be effectively harnessed in future transcriptomic studies to form a hierarchical validation pathway. For example, a large number of prioritized DEGs could be screened in relatively inexpensive and efficient traditional cell lines. Promising candidates from this work could then be investigated in more complex iPSC-RGC models to identify a small number of candidates for validation in animal models with a view towards translation. Furthermore, by using clinically demonstrated vectors (such as the adeno-associated virus [92]) candidate genes could be manipulated *in vivo*, with the advantage of being able to investigate meaningful effects at an organism level—for example, by quantifying visual acuity [93] or electrophysiological measures [33].

### 4.3. Therapeutic Development

#### 4.3.1. Personalized Medicine

The clinical application of transcriptomic techniques has increased our ability to provide a confirmed molecular diagnosis to patients with a suspected inherited optic neuropathy. In recent years, this has become particularly important as gene replacement therapies have advanced to human clinical trials, as in LHON [94,95]. Clinical transcriptomics are already in use to personalize therapy regimes in areas such as oncology [96], and its future application to inherited optic neuropathies represents an exciting development in the field.

#### 4.3.2. Transferable Neuroprotective Strategies

Transcriptomics has the great potential to identify targets for *generalizable*, mutation-independent neuroprotective strategies for inherited optic neuropathies. These paradigms of mitochondrial disease can provide a platform from which to identify and develop novel therapeutics for other ocular and neurodegenerative diseases in which mitochondrial dysfunction has been implicated. This is particularly timely, given not only the ongoing maturation of models with which to functionally validate transcriptomic findings in optic neuropathy, but also the concurrent explosion in innovative ocular gene therapy approaches and viral vector delivery [97,98,99]. Such techniques which are already licensed for clinical use in other conditions could be used to quickly develop therapies based on *in vitro* work to directly manipulate the intended therapeutic targets [92]. For example, “gene replacement” therapy has been successfully used to express wild-type copies of genes with homozygous null or haploinsufficient heterozygous pathogenic variants *in vivo* [92,100,101], with similar techniques also used to express neuroprotective genes such as brain-derived neurotrophic factor (BDNF) in animal models [98]. Additionally, techniques to downregulate genes with a dominant negative effect have also been developed and successfully applied to various inherited retinal disorders [102,103,104,105].

One of the reasons that *in vivo* ocular gene therapy has been so successful is the accessibility of the target cells, with RGCs being the first cell type encountered by therapeutic agents injected intravitreally (be it a viral vector, small molecule compound [106], or other biologic therapy [97,107,108]). Clearly, the extrapolation of potential neuroprotective strategies from an ocular model to more generalized central nervous system disorders will pose additional challenges. Here, direct access to tissue will not be as straightforward and much larger vector doses may be required, with an increased risk of adverse off-target effects [109], which will require close monitoring.

## 5. Conclusions

In this review, we have critically appraised how powerful transcriptomic techniques have the potential to facilitate the diagnosis of patients with inherited optic neuropathies and, crucially, achieve a better understanding of the pathological processes that contribute to RGC loss. The insight gained can then be exploited to develop targeted therapies to enhance RGC survival, which could have much broader relevance for other neurodegenerative diseases characterized by disturbed mitochondrial function. Presently, an unprecedented confluent maturation of complementary techniques is occurring in single-cell transcriptomics, bioinformatics, relevant disease models, and clinically translatable effector techniques. The potential for therapeutic advances in the eye, and beyond, is an exciting proposition for the coming decade.

## Figures and Tables

**Table 1:** Studies in the literature performing transcriptomic techniques in models of inherited optic neuropathy. Studies in glaucoma and mitochondrial disease in general have been reviewed [82]. LHON—Leber Hereditary Optic Neuropathy; *OPA1*-DOA—Dominant Optic Atrophy caused by *OPA1* mutations; RGC—Retinal Ganglion Cells; iPSC—induced pluripotent stem cells; KO—Knock Out; AAV—Adeno associated virus; GABA—γ-aminobutyric acid.

Reference	Condition	Model Used	Control	Technique	Conclusion
Danielson 2005 [83]	LHON	Cybrid cell lines containing mt11778 mutation	Cybrid cell lines without mt11778 mutation	Affymetrix U95Av2 oligonucleotide microarray	96 differently expressed genes, but only 9 of these replicated in other models.
Cortopassi 2006 [84]	LHON	Multiple cell lines carrying mt3460, 11778 or 14484 mutation for LHON as well as other cell lines corresponding to other common mitochondrial diseases	Corresponding cell lines without LHON causing mutations.	Affymetrix U95Av2 oligonucleotide microarray	Across models of multiple mitochondrial diseases, unfolded protein response and cell cycle pathways are upregulated and those involving vesicular secretion and protein synthesis are downregulated.
Yu 2015 [85]	LHON/complex I deficiency	Ndufs4 knock out mouse	Wild-type mice	Whole retina bulk RNA-seq	At whole retina level, genes most dramatically over represented are those associated with innate immunity and inflammation.
Cheng 2018 [86]	*OPA1*-DOA	Cultured RGCs derived from human stem cells with mutations in *OPA1* introduced by CRISPR	Cultured RGCs derived from human stem cells without mutations	scRNA-seq	Exploratory study. Pathways involved in RGC fate specification, axon guidance, and regeneration upregulated in *OPA1*-mutated cells.
Wu 2018 [87]	LHON	Cultured RGCs derived from iPSC from a LHON patient (mt11778 mutation)	Cultured RGCs derived from an asymptomatic carrier and control	GeneChip Human Genome U133 Plus 2.0 oligonucleotide microarrays	Genes implicated in “cell cycle” and extracellular matrix most over represented.
Calayan 2020 [88]	*OPA1*-DOA	Cultured neurons heterozygous for *OPA1* KO Cultured patient induced neural progenitor cells (c.2873_2876delTTAG)	Corresponding cells without mutation	Bulk RNA-seq	Downregulation of genes important for GABAergic neurons and retinal development.
Yu 2020 [89]	LHON	Optic nerve tissue from DBA1/J mice intravitreally injected with AAV coding for human *ND6* T14484C mutation	Optic nerve tissue from uninfected eyes	Bulk RNA-seq	Marked changes in gene expression, with pathways relating to oxidative stress and apoptosis particularly represented.
